# The Application of Existing Risk Assessment Models (RAMS) to Predict the Occurrence of Venous Thromboembolic Events among Patients with Classic Hodgkin Lymphoma

**DOI:** 10.3390/jcm13020436

**Published:** 2024-01-12

**Authors:** Mohammad Ma’koseh, Alaa Abufara, Dana Albaghdadi, Ruba Ghalayni, Sarah Abdel-Razeq, Eman Alzughali, Fadwa Abdel Rahman, Yazan Alhalaseh, Khalid Halahleh, Hikmat Abdel-Razeq

**Affiliations:** 1 Department of Internal Medicine, King Hussein Cancer Center, Amman 11941, Jordan; mm.09744@khcc.jo (M.M.); aa.10579@khcc.jo (A.A.); danabaghdadi95@gmail.com (D.A.); ruba.ghalayni@nm.org (R.G.); eman.eme211996@gamil.com (E.A.); yazanalhalaseh94@gmail.com (Y.A.); kh.06314@khcc.jo (K.H.); 2 School of Medicine, The University of Jordan, Amman 11942, Jordan; sar0191156@ju.edu.jo; 3 Department of Radiation Oncology, King Hussein Cancer Center, Amman 11941, Jordan; faelrahman@khcc.jo

**Keywords:** Hodgkin lymphoma, thrombosis, venous thromboembolic events, VTE, Khorana score, ThroLy score

## Abstract

**Background:** A majority of patients included in risk assessment models (RAMs) developed to predict venous thromboembolic events (VTE) in lymphoma were non-Hodgkin lymphoma. Our study aims to evaluate the incidence and predictors of VTE, utilizing different RAMs, in patients with classic Hodgkin lymphoma (cHL) treated with adriamycin, bleomycin, vinblastine, and dacarbazine (ABVD). **Methods**: Adult patients with cHL, treated and followed at our center, were included. Correlations between different variables, Khorana score, and thrombosis in lymphoma (ThroLy) RAMs with VTE were examined using Fisher’s exact test and logistic regression analysis. **Results**: A total of 321 patients were included, with a median age of 29 (range: 18–83) years. Of them, 169 (52.6%) had advanced-stage disease. Combined modality treatment was given to 169 (52.6%) patients. A total of 52 (16.2%) patients had relapsed or refractory disease. VTE were reported in 15 (4.7%) patients and were mostly during the administration of first-line (*n* = 8, 53.3%), or salvage chemotherapy (*n* = 6, 40.0%). There was no correlation between a Khorana score > 2 (*p* = 0.689) or ThroLy score > 3 (*p* = 0.335) and VTE. Older age (*p* = 0.014) and relapsed or refractory disease (*p* = 0.003) significantly correlated with VTE. **Conclusions**: VTE are uncommon in cHL. The commonly used RAMs failed to predict VTE. However, older age and relapsed or refractory disease significantly increased this risk.

## 1. Introduction

Venous thromboembolic events (VTE) are commonly encountered in patients with cancer [1]. Several studies have shown that lymphoma patients undergoing active chemotherapy are at an even higher risk of developing VTE [2,3]. Such episodes can be fatal, may delay chemotherapy and important invasive interventions, and may lead to immediate and delayed complications that may negatively affect the quality of life (QoL) of survivors [4].

Thromboprophylaxis for surgical and medical patients, including those with cancer, admitted to the hospital with acute medical problems or for surgical intervention is widely practiced and improving [5]. However, patients with cancer, even those receiving infusional chemotherapy, are treated in the outpatient settings. Many studies have shown that a majority of cancer patients develop their VTE while in ambulatory settings where VTE prophylaxis is not routinely practiced [6].

Lymphoma is classified among the high-risk cancer sites for development of VTE [7]. The incidence of VTE is lower in patients with classic Hodgkin lymphoma (cHL) than in non-Hodgkin lymphoma [8,9] with reported rates in the range of 3–20% [8,9,10,11,12].

Several risk assessments models (RAMs) for chemotherapy-associated thrombosis attempted to identify higher-risk patients for VTE [7,13,14,15]. Due to poor discriminatory performance, particularly when studied on a single cancer cohort, most such models have proven to be of limited clinical utility [16,17,18,19].

Given these limitations, Antic et al. developed and validated a new model, known as the thrombosis in lymphoma (ThroLy) score, to predict thromboembolic events in lymphoma patients [8,20]. Although it was validated in patients included in the German Hodgkin Study Group (GHSG) HD13–HD15 trials [21] and in two other studies [10,11], its clinical application is complex and failed to predict VTE in a follow-up study [9].

Moreover, in a comprehensive analysis of VTE in three GHSG HD13–HD15 trials, the incidence of VTE was higher in patients treated with dose-dense regimens [12]. 

Our study aims to evaluate the incidence and predictors, including commonly used RAMs of VTE in HL patients treated with adriamycin, bleomycin, vinblastine and dacarbazine (ABVD). 

## 2. Materials and Methods

Adult patients (≥18 years) with pathologically-confirmed cHL and diagnosed, treated, and followed-up at our institution from 2014 till 2019 were identified. Data were retrospectively collected from the hospital-based cancer registry and patients’ electronic medical records. 

Different clinical-, laboratory-, imaging-, and therapy-related variables including age, performance status (PS), personal history of VTE, myocardial infarction or stroke, smoking history, complete blood count, serum albumin, initial stage, mediastinal involvement, response to initial treatment, and salvage chemotherapy were collected. 

The disease stage and response to treatment were determined according to the Lugano criteria [22]. Patients with stage I or II were defined as early-stage disease, while patients with stage III and IV were defined as advanced-stage disease. Bulky disease was defined as a tumor mass of more than 10 cm on the initial CT scan. Please change this paragraph to: 

The Khorana RAM was calculated by adding one point attributed to the diagnosis of lymphoma to the platelet count ≥ 350 × 10^3^/µL (1 point) or hemoglobin level < 10 g/dL or using erythrocyte growth factors (1 point) or, leukocyte count > 11 × 10^3^/µL (1 point), and the body mass index (BMI) ≥ 35 kg/m^2^ (1 point). [7]. The ThroLy RAM score was calculated as previously described (Supplementary Table S1) [15].

No routine imaging studies to screen for VTE were performed. The diagnosis of VTE was radiologically confirmed with doppler ultrasound for deep vein thrombosis (DVT), and computed tomography (CT) angiography for pulmonary embolism (PE) for symptomatic patients. Details of thrombotic events were retrieved from radiology reports. 

VTE were considered chemotherapy-related if diagnosed any time after the first dose of chemotherapy and up to 4 weeks after the last, and were considered ambulatory if the patients were not admitted to the hospital during the 30 days prior to VTE diagnosis. Major bleeding, as a complication of anticoagulation, was defined as fatal bleeding and/or symptomatic bleeding in a critical organ and/or bleeding causing a fall in hemoglobin level of ≥2.0 g/dL, or leading to the transfusion of two or more units of red blood cells.

Descriptive statistics were used to characterize patients at the baseline. Continuous variables were presented as median (range), and categorical variables were expressed as numbers (percentages). Patients’ characteristics and VTE were compared using Fisher’s exact test for categorical variables and the Mann–Whitney U test for continuous variables. Multivariate logistic regression analysis was performed for significant variables in univariate analysis. Survival outcomes were calculated using the Kaplan–Meier method. The *p*-values are two-sided and considered significant when <0.05. Due to the retrospective nature of the study, written informed consent was waived and the data were anonymized and maintained with confidentiality. The study was approved by the King Hussein Cancer Center Institutional Review Board (IRB).

## 3. Results

### 3.1. Patients’ Characteristics

A total of 321 patients with a pathological diagnosis of cHL were included with a median age of 29 (18–83) years and almost equal gender distribution. At the time of diagnosis, only 11 (3.4%) patients had an Eastern Cooperative Oncology Group (ECOG) PS > 1, 169 (52.6%) patients had advanced-stage (stage III–IV) disease, 91 (28.3%) had bulky disease, and 250 (77.9%) had mediastinal involvement.

Most of our patients (*n* = 267, 83.2%) were treated with more than four cycles of ABVD. Consolidative radiotherapy was given to 169 (52.6%) patients, among which 127 (75.1%) had early-stage disease. After the completion of first-line treatment, 289 (90.0%) achieved a complete response (CR), 32 (10.0%) had refractory disease, and 20 (6.2%) had a relapse. Patients’ characteristics are detailed in Table 1.

### 3.2. Venous Thromboembolism

VTE were reported in 15 (4.7%) patients; a majority (*n* = 10, 66.7%) were ambulatory, and 11 (73.3%) were chemotherapy related. The median time from the diagnosis of cHL to the development of VTE was 6.9 (range: 0.3–42.1) months.

VTE were diagnosed most in the upper extremities (*n* = 12, 80.0%), followed by PE alone (*n* = 2, 13.3%) and the lower extremities (*n* = 1, 6.7%).

Among the upper-limb VTE, eight (66.7%) were right-sided, three (25%) were left-sided, and one (8.3%) was on both sides (extensive catheter-related thrombus). VTE location was the superior vena cava in five (41.7%) (all were catheter-related), in basilic, cephalic, cubital, and radial veins in four (33.3%), which was possibly related to chemotherapy, and three (25%) were possibly related to the compressive effects of lymph nodes: internal jugular vein compression caused by a mediastinal mass, supraclavicular vein compression caused by a supraclavicular mass, and axillary vein compression caused by an axillary mass.

At the time of VTE diagnosis, a majority (*n* = 13, 86.7%) had active disease, 11 (73.3%) had mediastinal involvement and only 5 (33.3%) patients had a central venous catheter (CVC).

VTE were treated with low-molecular weight heparin (LMWH) for a median duration of 6 (range: 3–12) months. While on anticoagulants, no episodes of major bleeding were reported. Recurrent VTE, however, were reported in one patient who developed PE four months after an upper-extremity DVT, while on therapeutic doses of LMWH.

### 3.3. Risk Factors for VTE

Among the continuous variables evaluated, age is the only variable that was significantly different between the group of patients with VTE compared to those without it (*p* = 0.011; Table 2). Among the categorical variables, failure to achieve CR after frontline treatment (*p* = 0.002) and no radiotherapy (*p* = 0.039) were found to be more frequent in patients with VTE (Table 3). In multivariate analysis, only older age (*p* = 0.014) and failure to achieve a CR after initial therapy (*p* = 0.004) were significantly associated with VTE (Table 4).

As 77.1% of VTE were reported in patients with mediastinal involvement (*n* = 250), we performed a separate analysis for potential variables that may correlate with VTE. In the univariate analysis, VTEs were more frequent in patients with refractory disease (*p* = 0.006), radiotherapy (*p* = 0.028), and age (*p* = 0.002). In multivariate analysis, refractory disease (*p* = 0.009) and age (*p* = 0.004) independently correlated with VTE.

### 3.4. Risk Assessment Models

According to the Khorana RAM, 266 (29.9%) patients had an intermediate-risk (score 1–2) and 55 (17.1%) others had a high-risk score (>2). Rates of VTE were not significantly different between the high-risk group (*n* = 2, 3.6%) and the intermediate-risk group (*n* = 13, 4.9%) (*p* = 0.689, Table 3). The Khorana risk score was also assessed as a continuous variable, and the mean score was not different between patients who had VTE vs. no VTE (1.67 vs. 1.78, *p* = 0.56; Table 2).

When patients were risk-stratified according to ThroLy RAM, 30 (9.3%) patients had a low-risk score (0–1), 198 (61.7%) patients had an intermediate-risk score (2–3), and 93 (29.0%) others had a high-risk score (>3). Although rates of VTE increased with higher-risk score, with *n* = 0 (0%), *n* = 9 (4.9%), and *n* = 6 (6.5%) in the low-risk, intermediate-risk, and high-risk groups, respectively, the difference was not statistically significant (*p* = 0.343; Table 3). In addition, we compared rates of VTE between patients with ThroLy risk scores ≤ 2 (*n* = 145, 45.2%) vs. >2 (*n* = 176, 54.8%), and there was no significant difference between the two groups (VTE *n* = 5, 3.4% vs. 10, 5.7%, *p* = 0.335; Table 3).

Even when the ThroLy risk score was evaluated as a continuous variable, the mean ThroLy score was higher in patients with VTE compared to those with no VTE (3.4 vs. 2.7); however, the difference was not statistically significant (*p* = 0.07; Table 2).

### 3.5. Survival Outcomes

**Figure 1:**
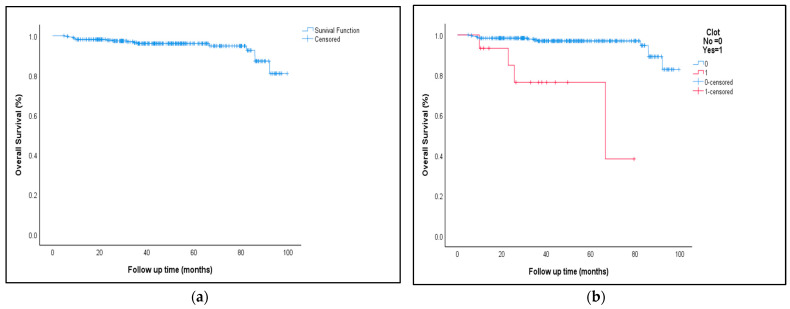
Overall survival (OS) for all patients enrolled (**a**), and in relation to the presence or absence of VTE (**b**).

At a median follow-up of 45 (range: 5–100) months, the five-year overall survival (OS) rate for the whole group was 96% (Figure 1a). However, compared to patients with no VTE, patients with VTE had a significantly lower OS (78% versus 97%, *p* < 0.001; Figure 1b).

## 4. Discussion

This study provides additional evidence that the universal application of RAMs used to predict VTE may not be suitable in a specific cancer. Even among lymphomas, different lymphoma subtypes have significant differences in the incidence and predictors of VTE. Patients with cHL were underrepresented in most studies that evaluated the risk of VTE in lymphoma. For example, only 15% of patients included in the Antic et al. study had cHL [8,20]. Moreover, when the ThroLy RAM was validated in cHL treated in the GHSG HD13–HD15 trials [21], the validation cohort included patients treated with heterogeneous chemotherapeutic regimens according to the trial design.

The overall rate (4.9%) and the median time from diagnosis to VTE (6.9 months) in our study are almost similar to what was reported in most previous studies and a meta-analysis [3,8]. In addition, the incidence of VTE in 6.9% in patients with advanced-stage disease is in line with high-risk cancer patients defined by Khorana et al. [7].

Most of the VTE reported in our study were in ambulatory patients, having active disease and while receiving chemotherapy, which is consistent with the previous studies about VTE in cancer patients [6] as well lymphoma patients [3]. More interestingly, 80% of encountered VTE were in the upper extremity (UEDVT); only 5, less than 50%, could be attributed to CVC. The predominance of UEDVT is consistent with the previous reports [10,12]. Factors that may have contributed to the development of UEDVT were the presence of CVC, irritation and inflammation related to chemotherapy administration in the upper limbs, and compressive effects of enlarged lymph nodes.

Although patients older than 50 years represent only 6.5% of our patients, older age, when evaluated as a continuous variable, was independently associated with increased risk for VTE. Borchmann et al. analyzed the thrombotic events in cHL treated in GHSG HD13–HD15 trials that excluded patients older than 60 years in the HD14 and HD15 trials, and reported a similar age association, with almost identical odds ratios, of 1.047 and 1.02, respectively [12].

In our study, failure to achieve a complete response after frontline treatment, which indicates the over-all aggressiveness of the disease and the need for more intensive, platinum-based salvage chemotherapy, had the strongest correlation with VTE. On the other hand, other variables previously reported to be associated with VTE in lymphoma including advanced-stage disease, mediastinal involvement, extranodal involvement, and a higher number of chemotherapy cycles, had no correlation. This can be explained by the relatively low number of VTE, and the fact that the achievement of complete response in cHL is a robust indicator of survival that outperforms other clinical and laboratory variables [23].

Failure of the Khorana RAM to predict VTE in cHL was also reported in two other studies [11,12]. According to the Khorana RAM, lymphoma patients are either classified as intermediate- or high-risk, and this may result in poor discrimination between the two groups. Interestingly, we found that 13 out of 15 (86.6%) VTE were reported in the intermediate-risk group, which is in line with the results of a recent meta-analysis that included 27,849 cancer patients and found that 76.6% of VTE were reported outside the high-risk group [24].

The original ThroLy RAM included a diverse group of lymphoma patients, of whom most were NHL, who are at a higher risk of VTE. Similar to the Khorana RAM, the score performance is limited by the high number of VTE outside the high-risk group [25]. In the validation study by Rupa-Matyesk et al., 48% of VTE were reported in the low-risk group, among which cHL represented 61% of patients [9]. In our study, 9 out 15 (60%) VTE were reported outside the high-risk group. Moreover, when the ThroLy RAM was validated in the GHSG HD13–HD15 trials, its sensitivity and specificity were 45% and 65%, respectively [21]. 

Although the ThroLy RAM includes variables that are more relevant to predict VTE in lymphoma patients, a previous history of VTE (2 points), reduced mobility (1 point), a previous history of myocardial infarction or stroke (2 points), and neutropenia (1 point), representing 6 out of 12 possible points, are rare in cHL patients because they are generally younger and rarely have pre-existing comorbidities. The limited contribution of these variables to the ThroLy score in cHL, added to the weak association of other variables with VTE, may have contributed to the lack of a significant association between the ThroLy score and VTE in our study. 

We previously validated the ThroLy RAM in diffuse large B cell lymphoma (DLBCL) [26]. The main components of the ThroLy RAM that were associated with VTE were a previous history of thrombosis, reduced mobility, and low hemoglobin. Compared to DLBCL patients included in the validation study, cHL patients included in this study had a relatively lower frequency of these components (3.1% vs. 8.6% and 3.4% vs. 16.2%, respectively), possibly because the median age of DLBCL patients was 20 years older (49 vs. 29 years). In addition, the lower incidence of VTE in cHL (4.7% vs. 13.5%) may have contributed to the lack of a significant correlation between the ThroLy score and VTE in cHL.

Cancer-associated thrombosis is a leading cause of death in cancer patients [4,27]. Previous studies in lymphoma confirmed the negative impact of VTE on mortality [10,28]. The increased mortality rate in patients with VTE in our study can be explained by their association with the refractory disease, which is known to be associated with worse survival in cHL. The abundant cellular, humoral, and inflammatory infiltrates in cHL play a pivotal role in enhancing the survival of Reed–Sternberg cells, resulting in a lack of response to treatment [29]. Local tumor inflammation has been implicated in the pathophysiology of VTE in cHL as well [12], and may explain the association of VTE with a lack of response to treatment and increased mortality. 

The retrospective design of the study, and the inclusion of patients from a single center, are among the study limitations. However, the inclusion of a relatively high number of cHL patients treated with a uniform chemotherapy regimen, commonly used in the frontline treatment of cHL, adds to the value of our study.

## 5. Conclusions

Khorana and ThroLy RAMs failed to predict VTE in cHL treated with ABVD. Older age and a lack of achievement of a complete response to frontline treatment were the main predictors of VTE. Such patients may be considered for prophylactic anticoagulation in a prospective study.

## Figures and Tables

**Table 1:** Patients’ characteristics.

Characteristic		Number	Percentage
Age (years)	Median Range	29 18–83	
Gender	Male Female	163 158	50.8 49.2
ECOG performance status	>1 0–1	11 310	3.4 96.6
BMI (kg/m^2^)	BMI > 30 BMI > 35	93 36	29.0 11.2
Smoking history	Current smoker Prior smoker Never smoked	130 25 176	37.4 7.8 54.8
Prior VTE	Yes No	10 311	3.1 96.9
Histology	Nodular sclerosis Mixed cellularity Lymphocyte-depleted Lymphocyte-rich Classical (NOS)	243 44 1 2 31	75.7 13.7 0.3 0.6 9.7
Staging	Bulky disease Mediastinal involvement Extranodal involvement Early stage Advanced stage Stage IV B-symptoms	91 250 119 152 169 75 174	28.3 77.9 37.1 47.4 52.6 23.4 54.2
Blood count	Hemoglobin < 10 gm/dL WBC count > 11 × 10^3^/µL Platelet count > 350 × 10^3^/µL Neutrophil count < 1 × 10^3^/µL	57 137 156 3	17.8 42.7 48.6 0.93
Serum albumin	Low Normal	116 205	36.1 63.9
Number of ABVD cycles	2–4 cycles >4 cycles	53 267	16.5 83.2
Radiotherapy	Consolidation No consolidation	169 152	52.6 47.4
Response to initial treatment	Complete response Refractory	289 32	90.0 10.0
Salvage chemotherapy	Yes No	39 282	12.1 87.9
Khorana score	>1 >2	191 55	59.5 17.1
ThroLy score	0–1 2–3 >3 0–2 >2	30 (9.3%) 198 93 145 176	9.3 61.7 29.0 45.2 45.8

ECOG: Eastern Cooperative Oncology Group; BMI: body mass index; VTE: venous thromboembolism; NOS: not otherwise specified; WBC: white blood cells; ABVD: adriamycin, bleomycin, vinblastine, and dacarbazine.

**Table 2:** Correlation between continuous variables and VTE.

Variable	Patients with No VTE (Mean)	SD	Patients with VTE (Mean)	SD	*p*-Value	95% CI
Age (years)	30.8	11.21	38.4	14.95	0.012	1.69–13.56
BMI (kg/m^2^)	26.8	6.07	28.3	6.05	0.33	−1.6–4.7
Hemoglobin (gram/dL)	12.2	2.24	12.5	1.98	0.58	−1.85–4.94
WBC count (×10^3^/µL)	11.5	6.2	12	4.57	0.78	−2.67–3.53
Neutrophil count (×10^3^/µL)	7.4	5.78	9.2	4.78	0.22	−1.13–4.83
Lymphocyte count (×10^3^/µL)	1.7	0.82	1.4	0.63	0.13	−0.75–0.097
Platelet count (×10^3^/µL)	362	134.8	369	147.5	0.845	−63.4–77.4
Albumin (gram/dL)	4.1	0.54	3.8	0.49	0.095	−0.52–0.042
ESR (mm/hour)	60.5	38.6	68	38.75	0.49	−14.07–29
Khorana score	1.78	0.75	1.67	0.72	0.56	−0.5–0.27
ThroLy score	2.7	1.3	3.4	1.3	0.07	−0.05–1.3

VTE: venous thromboembolism; SD: standard deviation; CI: confidence interval; BMI: body mass index; WBC: white blood cell; ESR: erythrocyte sedimentation rate.

**Table 3:** Univariate analysis of categorical variables.

Characteristic		No VTE	VTE	*p*-Value
Gender	Male Female	50.7% 49.3%	53.3% 46.7%	0.839
ECOG performance status (PS)	≤1 >1	96.7% 3.3%	93.3% 6.7%	0.481
BMI (kg/m^2^)	≤30 >30 <35 ≥35	71.6% 28.4% 89.2% 10.8%	60% 40% 80% 20%	0.335 0.269
Smoking history	Current/prior smoker Never smoked Current smoker Prior smoker/never smoked	44.1% 55.9% 36.6% 63.4%	66.7% 33.3% 53.3% 46.7%	0.087 0.191
Prior thrombosis	Yes No	2.9% 97.1%	6.7% 93.3%	0.417
B-symptoms	No Yes	53.1% 46.9%	40% 60%	0.645
Histology	Nodular sclerosis Mixed cellularity Lymphocyte-depleted Lymphocyte-rich Classical (NOS)	75.% 14.4% 0.3% 0.7% 9.2%	80% 0% 0% 0% 20%	0.395
Bulky disease	Non-bulky Bulky	71.9% 28.1%	66.7% 33.3%	0.661
Mediastinal involvement	No Yes	22.9% 77.1%	6.7% 93.3%	0.14
Extranodal involvement	No Yes	63.7% 36.3%	46.7% 53.3%	0.182
Stage	Early stage (I/II) Advanced stage (III/IV)	48% 52%	33.3% 66.7%	0.265
	Stage I–III Stage IV	69.9% 30.1%	46.7% 53.3%	0.057
Hemoglobin (gram/dL)	>10.5 <10.5 ≥10 <10	75.8% 24.2% 81.4% 18.6%	93.3% 6.7% 100% 0%	0.118 0.065
WBC (×10^3^/µL)	WBC count ≤ 11 WBC count > 11	58.2% 41.8%	40% 60%	0.165
Platelet count (×10^3^/µL)	Platelet count < 350 Platelet count ≥ 350	51.3% 48.7%	53.3% 46.7%	0.878
Neutrophil count (×10^3^/µL)	Neutrophil count ≥ 1 Neutrophil count < 1	99% 1%	100% 0%	0.7
Serum Albumin	Normal Low albumin	64.4% 35.6%	53.3% 46.7%	0.385
Number of ABVD cycles	2–4 cycles >4 cycles	18% 82%	26.7% 73.3%	0.396
Radiotherapy	No radiotherapy Radiotherapy	46.1% 53.9%	73.3% 26.7%	0.039
Response to initial therapy	No relapse or refractory disease Relapsed or refractory disease	85.6% 14.4%	53.3% 46.7%	0.001
BMT	No BMT BMT	88.6% 11.4%	73.3% 26.7%	0.078
Khorana score	0–1 >1 0–2 >2	40.2% 59.8% 82.7% 13.3%	46.7% 53.3% 86.7% 13.3%	0.618 0.689
ThroLy score	0–1 2–3 >3 0–2 >2	9.8% 61.8% 28.4% 45.8% 54.2%	0% 60% 40% 33.3% 66.7%	0.343 0.345

ECOG PS: Eastern Cooperative Oncology Group performance status; BMI: body mass index; WBC: white blood cell; ABVD: adriamycin, bleomycin, vinblastine, and dacarbazine; BMT: bone marrow transplant.

**Table 4:** Multivariate analysis.

Effect	Odds Ratio (OR)	95% Confidence Interval (CI)	*p*-Value
Age	1.047	1.009–1.087	0.014
Relapsed/refractory disease	5.614	1.783–17.681	0.003
No radiotherapy	2.004	0.587–6.847	0.267

## Data Availability

The data presented in this study are available upon request from the corresponding author.

## Supplementary Materials

The following supporting information can be downloaded at: https://www.mdpi.com/article/10.3390/jcm13020436/s1, Table S1: ThroLy risk assessment model.
