# Supplementary material for: The Application of Existing Risk Assessment Models (RAMS) to Predict the Occurrence of Venous Thromboembolic Events among Patients with Classic Hodgkin Lymphoma
Source: J Clin Med. 2024 Jan 12;13(2):436. doi: 10.3390/jcm13020436 (PMC10816014; doi:10.3390/jcm13020436)
Supplement: Supplementary file 1 [file jcm-13-00436-s001.zip › Table S1.pdf]

Supplementary Table S1: ThroLy Risk Assessment Model

**Patients' characteristics**	**Assigned score**
Previous venous thromboembolic event	2
Reduced mobility (ECOG 2-4)	1
Previous acute myocardial infarction or stroke	2
Body Mass Index > 30 Kg/m^2	2
Extranodal localization	1
Mediastinal involvement	2
Neutropenia	1
Hemoglobin < 10 grams/dL	1

Low risk (0-1), Intermediate risk (2-3), High risk (> 3)

ECOG: Eastern Cooperative Oncology Group; BMI: Body mass index.
